# Recognizing the role of Epstein-Barr virus in gastric cancer: transcriptomic insights into malignancy modulation

**DOI:** 10.1186/s12985-024-02307-z

**Published:** 2024-02-14

**Authors:** Tabassom Sedaghat Anbouhi, Hossein Sazegar, Ebrahim Rahimi

**Affiliations:** 1 Department of Biology, Faculty of Basic Sciences, Shahrekord Branch, Islamic Azad University, Shahrekord, Iran; 2 Department of Food Hygiene, Faculty of Veterinary Medicine, Shahrekord Branch, Islamic Azad University, Shahrekord, Iran

**Keywords:** RT-qPCR, Gene expression, In silico, TCGA, Survival rate, Virus

## Abstract

**Background:**

Studies show that Epstein-Barr virus (EBV) infection can play a role in malignancy and increase the risk of gastric cancer (GC). The objective of this research was to pinpoint genes whose expression may be influenced by EBV and play a role in the development of GC.

**Methods:**

Candidate genes potentially susceptible to expression modulation in the presence of EBV were identified through the analysis of GSE185627 and GSE51575 datasets. The association of candidate genes with GC and the survival rate of patients was investigated based on the cancer genome atlas (TCGA) data. Also, pathways related to candidate genes were examined through the MsigDB database. The PPI network was used to identify Hub genes. To corroborate the obtained results, we utilized the RT-qPCR method, employing GC samples from both EBV + and EBV-cases, as well as adjacent normal samples.

**Results:**

Our results showed that genes upregulated by the EBV in the GC cell line, as well as in EBV + samples, are significantly linked to pathways involving the immune response, inflammation, and the P53 pathway. Conversely, genes downregulated by EBV are closely linked to pathways involving cell proliferation and mTORC1. Examining the candidate genes revealed that a considerable portion of genes susceptible to downregulation under the influence of EBV exhibit oncogenic roles based on TCGA data. Moreover, some of these genes are associated with an unfavorable prognosis. Protein-protein interaction network analysis of candidate genes highlighted *IFI44L* and *OAS2* as potential hub genes in the EBV-GC axis. Our RT-qPCR results further validated these findings, demonstrating that the expression levels of *IFI44L* and *OAS2* were higher in EBV + samples compared to both healthy and EBV-samples.

**Conclusion:**

Our study underscores the capacity of EBV to exert regulatory control over genes associated with GC malignancy. In addition to its inflammatory effects, EBV elicits transcriptomic changes that appear to attenuate the progression of GC.

**Supplementary Information:**

The online version contains supplementary material available at 10.1186/s12985-024-02307-z.

## Background

Gastric cancer (GC) remains a substantial global health issue, with over one million new cases reported in 2020 and an estimated 769,000 associated deaths [1]. Molecular studies related to this disease have shown that, like other cancers, heterogeneity at the molecular level is a common phenomenon in this disease and can be a key factor in different treatment responses [2]. Therefore, molecular studies related to this cancer are still being conducted to identify new therapeutic and diagnostic targets.

Epstein-Barr virus (EBV)-associated GC is a common malignancy associated with EBV infection [3]. Studies have indicated that EBV can elevate mutation rates in genes like *PIK3CA* and *ARID1A*, consequently heightening the predisposition to GC [4]. Furthermore, it has been observed that samples testing positive for Epstein-Barr virus (EBV+) display a distinct DNA methylation profile. This profile has the potential to influence the malignancy of GC by altering the states of gene expression [5]. Notably, research has revealed that patients with EBV + samples tend to enjoy a more favorable prognosis in contrast to their EBV-negative (EBV-) counterparts [6]. These results highlight the diverse impact of EBV in GC, increasing susceptibility while also providing a more optimistic outlook for survival and treatment response in the EBV + subgroup. Thus, it becomes imperative to discern the molecular mechanisms intricately connecting EBV contamination and the pathogenesis of GC.

Although various studies have shown that EBV can increase the risk of GC and is present in approximately 10% of GC samples [7], the mechanisms of related gene expression changes have been less investigated. Also, the relationship between the genes affected by EBV and the prognosis of patients has been less investigated. The purpose of this study was to identify genes that can undergo expression changes under the influence of EBV. Then, the relationship of the identified candidate genes with the pathways related to GC and the association of their expression with the mortality rate of patients were investigated. Finally, by using the PPI network, hub genes related to EBV were identified, and their expression levels were investigated in EBV + samples compared to normal and EBV-samples.

## Materials and methods

### Data sources and preprocessing

To identify genes related to EBV in GC, a Gene Expression Omnibus (GEO) study with accession number GSE185627 was used [8]. In this study, a GC cell line called AGS was used, and its transcriptome changes under the influence of EBV infection were investigated using the RNAseq method. This study had three control samples and three samples infected with EBV. The data of this study was downloaded in raw form, and using the edgeR package, genes with zero or close to zero expression based on the CPM (count per million) criterion of less than 10 in 70% of the samples were removed [9]. Then, based on the TMM (Trimmed Mean of M-values) method, the data were normalized, and the data was converted into a logarithmic form based on base 2 [10]. The resulting expression matrix was used for analysis. GSE51575 was also used to further confirm the results [11]. The data for this study was downloaded in raw format, and the initial pre-processing was done on the data, including background correction, normalizing the data based on the RMA method, and transferring the data to logarithmic mode based on 2 [12]. The resulting expression matrix was used for all analyses. GSE51575 included 12 EBV + samples and adjacent normal samples, as well as 14 EBV- samples with adjacent normal. The cancer genome atlas (TCGA) data were also used to investigate the relationship between the expression of candidate genes and GC. The transcriptomic data (RNAseq) related to GC (TCGA-STAD) was downloaded in raw format by the TCGAbiolinks package [13]. Genes with zero or close to zero expression based on the CPM criterion of less than 10 in 70% of the samples were removed. Then, based on the TMM method, the data were normalized and converted into a logarithmic form based on base 2. The resulting expression matrix was used for all analyses of expression differences and survival. GC samples in the TCGA database included 32 normal samples and 375 tumor samples.

### Calculation of gene expression differences

Samples from GSE185627 were categorized into two groups: control and those infected with EBV. The disparity in expression between these groups was computed using the linear model method [14]. The obtained genes were considered candidate genes that could be affected by EBV. Also, GSE51575 samples were divided into EBV- and EBV + groups, and the expression difference between these two groups was calculated. Finally, the TCGA data were divided into two cancer and normal groups based on the clinical data, and the difference in gene expression of between these two groups was calculated using the linear model method. A single method was used for all differences in expressions to avoid errors.

### Survival analyses

TCGA clinical data were used to identify the relationship between the expression of candidate genes and the survival of GC patients. For this purpose, patients whose number of days to live was NA, 0, and 1 were excluded from the study. Also, for samples with a dead status, only those that had tumors at the time of death were considered. After the applied filters, 250 samples were left, of which 51 were dead and 199 were alive for the survival test. Also, the normalized matrix was used to associate the expression of candidate genes with the survival of patients. In this regard, first, the expression of each gene in all samples was scaled (Z-score), and then the Z-score of each sample was integrated with clinical data. Finally, using the Cox regression test, the relationship between the expression of candidate genes and the mortality rate of patients was evaluated. To confirm the obtained results, the Kaplan-Meier (K-M) curve was used, and the median expression of candidate genes in cancer samples was used to divide the groups into two groups, high and low.

### Protein-protein interaction network

To identify the central genes affected by EBV, a protein-protein interaction (PPI) network was used. For this purpose, the interaction of all candidate genes was investigated using the string-db database (https://string-db.org/). The Cytoscape program (V 3.7.2) was used to create a PPI network between candidate genes. In addition, the molecular complex detection plugin (MCODE) was used to cluster the PPI network based on the following criteria: degree cut = 2, node score cut = 0.2, k-core = 2, and max. depth = 100. Finally, CytoHubba another plugin in Cytoscape for hub genes was used with 11 topological analysis methods to rank nodes in a network. In this study, Gene Topi classifies his accounts using the maximal clique centrality (MCC) method. Finally, hub genes were selected based on the mentioned method.

### Collection of samples

A total of forty GC samples and an equivalent number of adjacent normal tissue samples were meticulously collected from the repository of the Iranian Tumor Bank. The samples were fresh and stored in liquid nitrogen until use. Stringent ethical protocols were adhered to, and the study received the requisite approval from the review board of Imam Khomeini Hospital, the institution of origin for the samples. This ethical clearance was granted in strict accordance with the regulations stipulated by the Ministry of Health, Treatment, and Medical Education of Iran. Furthermore, the participation of candidates in this study was contingent upon their informed consent, and the research protocol was vetted and sanctioned by the ethics committee, bearing the reference number IR.IAU.PS.REC.1401.320. Each cancer sample underwent meticulous pathological examination to affirm its disease status, and a summary of the pertinent clinical details is succinctly presented in Table 1. The EBV infection status of each sample was comprehensively confirmed through pathological data analysis. The methods used to confirm EBV + samples were RT-qPCR and Epstein-Barr encoding region (EBER) in situ hybridization.

**Table 1:** Clinical information for GC samples

characteristic	Number (*N*=40)
Age	
<50 >50	17 23
Gender	
Male Female	22 18
Stage	
I II III IV	4 17 11 8
TNM.N	
N0 N1 N2 N3	7 16 10 7
Tumor size	
<5 cm >5 cm	16 24
Epstein-Barr	
EBV+ EBV-	14 26

### RNA extraction, cDNA synthesis, and RT-qPCR

The tissue samples underwent a thorough washing, involving three rinses with PBS-, to eliminate potential contaminants and necrotic cells. Subsequently, RNA extraction was carried out employing TRIzol (Genius Gene), in strict adherence to the manufacturer’s provided instructions. To further ensure the purity of the extracted RNA, DNase I treatment (Sina Clone) was administered. The subsequent step involved cDNA synthesis, which was executed following the precise guidelines furnished by the Genius Gene kit. For the specific genes *IFI44L* (F: 5’-CTTTTGTTCGTTTTGCCTTCTGT-3’ and R: 5’-CCCACCGCTTCTCAGGTTT-3’) and *OAS2* (F: 5’-GCTTCCGACAATCAACAGCCAAG-3’ and R: 5’-CTTGACGATTTTGTGCCGCTCG − 3’), primer sequences were designed using the primer-blast tool available through NCBI. Subsequently, the RT-qPCR method, in conjunction with the aforementioned specific primers and SYBR Green master mix (Genius Gene), was employed to quantitatively assess the expression levels of these designated genes within both cancer and normal tissue samples. The expression levels of each gene in each sample were computed utilizing the 2^−ΔCt^ method, with *GAPDH* serving as the internal reference control [15].

### Statistics and software

All pre-processing and data analysis were done by R programming (V 4.0.2) and GraphPad software (V 8) was used to draw and display graphs. The linear model method was used to calculate the difference in expression and the significance level between the groups was calculated through multiple hypothesis testing. FDR < 0.05 level was considered in all analyses. To check the significance level, the log-Rank test was used to check the relationship between the expression of candidate genes and the prognosis of patients. Cytoscape (V 4) was used to show the PPI network and the relationships between genes.

## Results

### Expression changes of many genes related to immune response and cell cycle under the influence of EBV

**Fig. 1:**
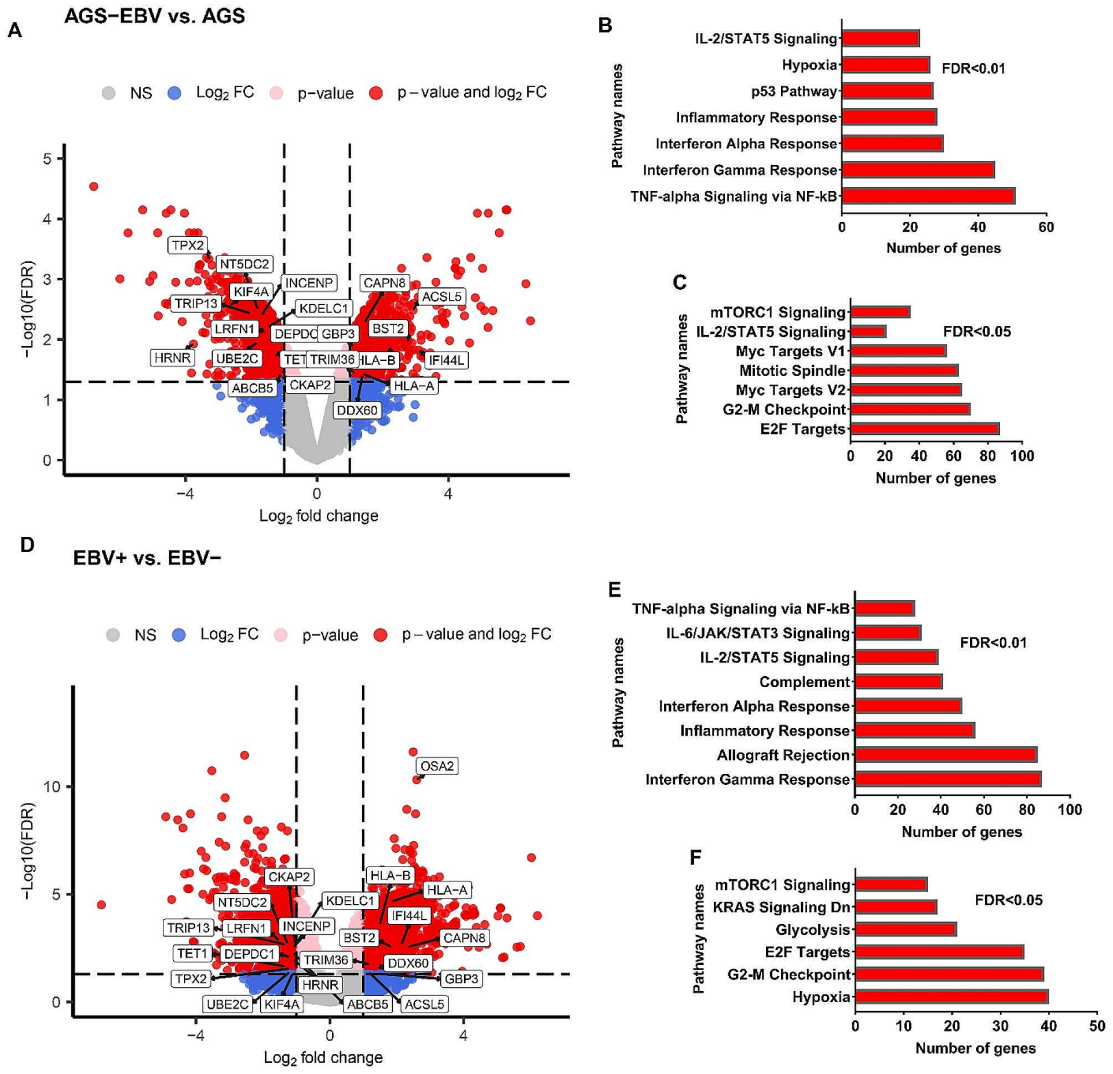
The Relationship Between EBV-induced gene expression changes and key malignancy pathways. (**A**) A volcano plot for all genes altered in a GC cell line due to EBV infection based on GSE185627 data is show. A fold change in expression greater than 1 or less than − 1, coupled with a FDR less than 0.05, was deemed as the cutoff criteri. (**B**) Enrichment results are shown for all genes that are increased due to EBV infection in GC cell lines. (**C**) Pathway results for genes that could be downregulated under the influence of EBV are highlighted. (**D**) The expression differences for all genes that exhibited changes in expression between EBV + and EBV- samples are depicted based on GSE51575. The cutoff criteria were defined as an expression fold change of larger than 1 or less than − 1 and an FDR < 0.05. (**E**) The pathways associated with upregulated genes in EBV + samples compared to EBV- samples are displayed. (**F**) The findings related to the enrichment of downregulated genes in EBV + samples compared to EBV- are presented

The genes that could be changed under the influence of EBV based on the GSE185627 study were examined. Analysis of expression differences showed that 931 genes had increased expression in the AGS-EBV cell line compared to AGS (Fig. 1A, logFC > 1 and FDR < 0.05). Conversely, 1122 genes were identified with a significant downregulation in expression (logFC<-1 and FDR < 0.05) under the influence of EBV (Fig. 1A). For more insight into the identified genes, the pathways associated with them were investigated. Enrichment results for 931 genes showed their involvement in pathways related to immune responses to viruses, p53, hypoxia, and inflammation. (Fig. 1B, FDR < 0.01). On the other hand, it was found that the down-expressed genes are involved in the main pathways related to cancer, such as cell proliferation, mTORC1, and cell division (Fig. 1C, FDR < 0.05). These results show that genes affected by EBV could have a negative effect on the proliferation of GC cells. To further confirm the obtained results, gene expression changes in virus-infected GC samples (EBV+) compared to non-infected GC samples (EBV-) were investigated using GSE51575. The results revealed that, in EBV + samples compared to EBV-, the expression of 1280 genes was significantly increased (Fig. 1D, LogFC > 1 and FDR < 0.05), while 1152 genes exhibited a significant decrease in expression (Fig. 1D, LogFC<-1 and FDR < 0.05). Genes that were upregulated in EBV + samples were associated with pathways related to inflammation and immune response to viruses (Fig. 1E, FDR < 0.01). On the other hand, downregulated genes in EBV + samples were linked to pathways involving hypoxia, cell proliferation, and mTORC1 (Fig. 1F, FDR < 0.05). These results suggest that, despite the inflammatory effects of EBV, it may have a mitigating impact on key cancer-related genes, particularly those associated with cell proliferation.

### Exploring the intersection of EBV-influenced genes and GC pathogenesis

**Fig. 2:**
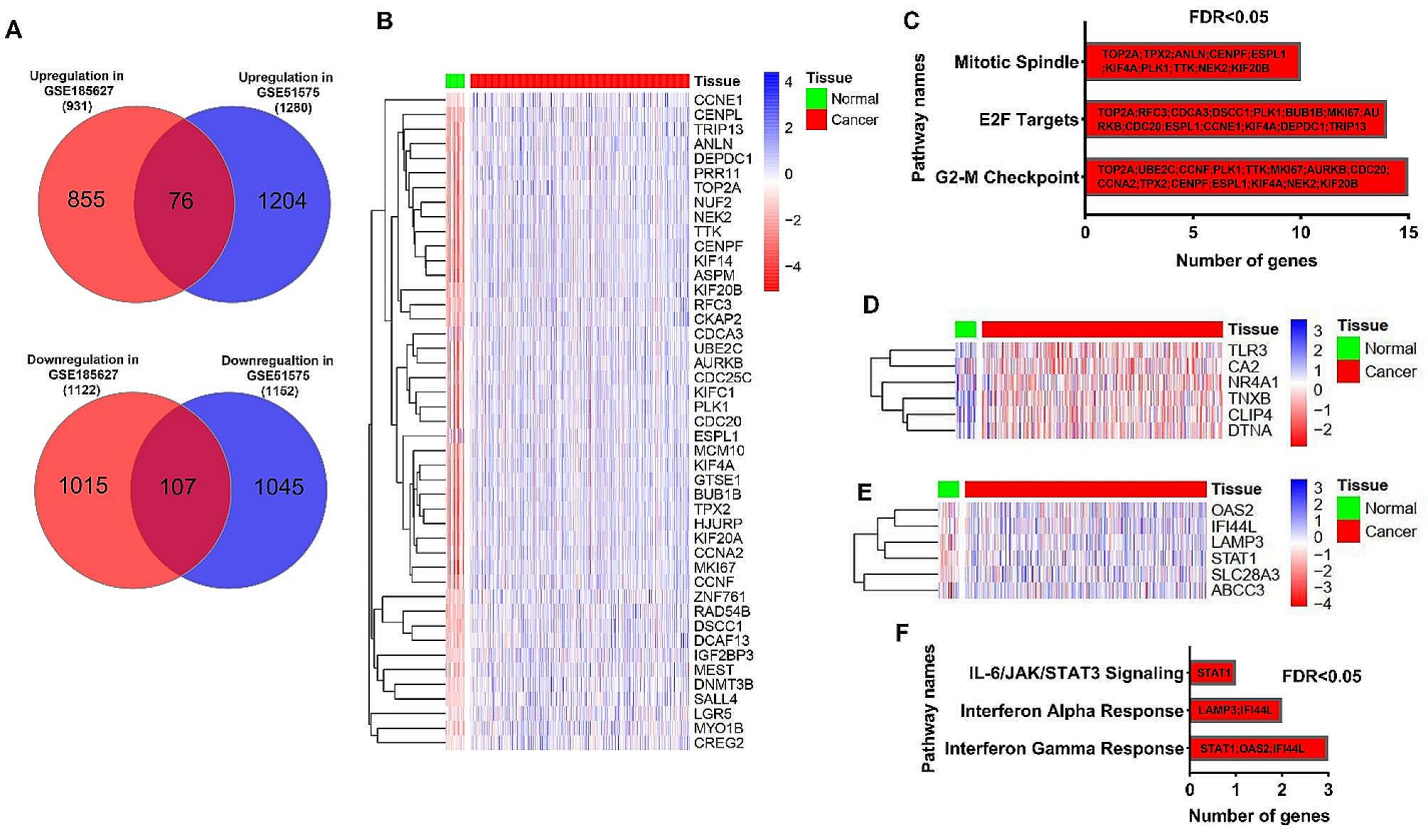
The significant alterations in the expression of a large number of genes influenced by EBV in GC based on the TCGA data. (**A**) A Venn diagram depicting the shared genes is displayed, highlighting the overlap between the results of gene expression differences observed in the treatment group with EBV in the gastric cancer cell line and the comparison between EBV + and EBV- samples. (**B**) A collection of genes, chosen as candidate genes, are indicated to exhibit increased expression in GC but are capable of undergoing downregulation under the influence of EBV infection. (**C**) The enrichment results for the genes present in the heatmap of B section are demonstrated. (**D**) The heatmap corresponds to six genes that exhibited decreased expression in GC while could potentially undergo increased expression under the influence of EBV load. (**E**) The expression changes of six identified genes in the TCGA data, which show increased expression in both GC samples and could also be upregulated by EBV, are depicted. (**F**) The pathway enrichment results for the genes present in section E is demonstrated

We analyzed TCGA data to explore the connection between genes susceptible to changes under EBV influence and genes associated with the development of GC. For this purpose, as shown in Figs. 2A and 76 genes were identified that could be increased in expression under the influence of EBV in both the GSE185627 and GSE51575 studies. Also, 107 genes were identified that can have a significant decrease in expression under the influence of EBV in both of the mentioned studies (Fig. 2A). These identified genes were designated as candidate genes associated with EBV and subjected to further detailed analysis, with their respective findings summarized in Table S1. The analysis of candidate gene expression changes in TCGA data revealed that 45 genes in GC samples exhibited a significant increase in expression (Fig. 2B, logFC > 1 and FDR < 0.01). Interestingly, these 45 genes displayed a simultaneous decrease in expression under the influence of EBV (Supplementary file, Fig. S1). Enrichment results for these 45 genes indicated that they are involved in pathways such as cell proliferation (Fig. 2C, FDR < 0.05). Six genes were identified in GC samples with a significant decrease in expression compared to normal tissue (Fig. 2D, logFC<-1 and FDR < 0.01). However, based on the previous results, these 6 genes could increase their expression under the influence of EBV. No significant pathway was identified for them. In contrast, the same behavioral genes were also evaluated. The results showed that 6 genes are significantly increased in cancer samples compared to normal ones (logFC > 1 and FDR < 0.01) and could be increased by EBV (Fig. 2E). The enrichment results of these 6 genes showed that they are involved in immune pathways such as interferon-gamma and alpha (Fig. 2F, FDR < 0.05). No genes were identified that were significantly down-regulated in TCGA data and could be down-regulated by EBV. These results suggest that genes associated with cancer promotion, especially those involved in cell proliferation, may be downregulated due to the effect of EBV.

### The relationship between genes affected by EBV and the mortality rate of patients

**Fig. 3:**
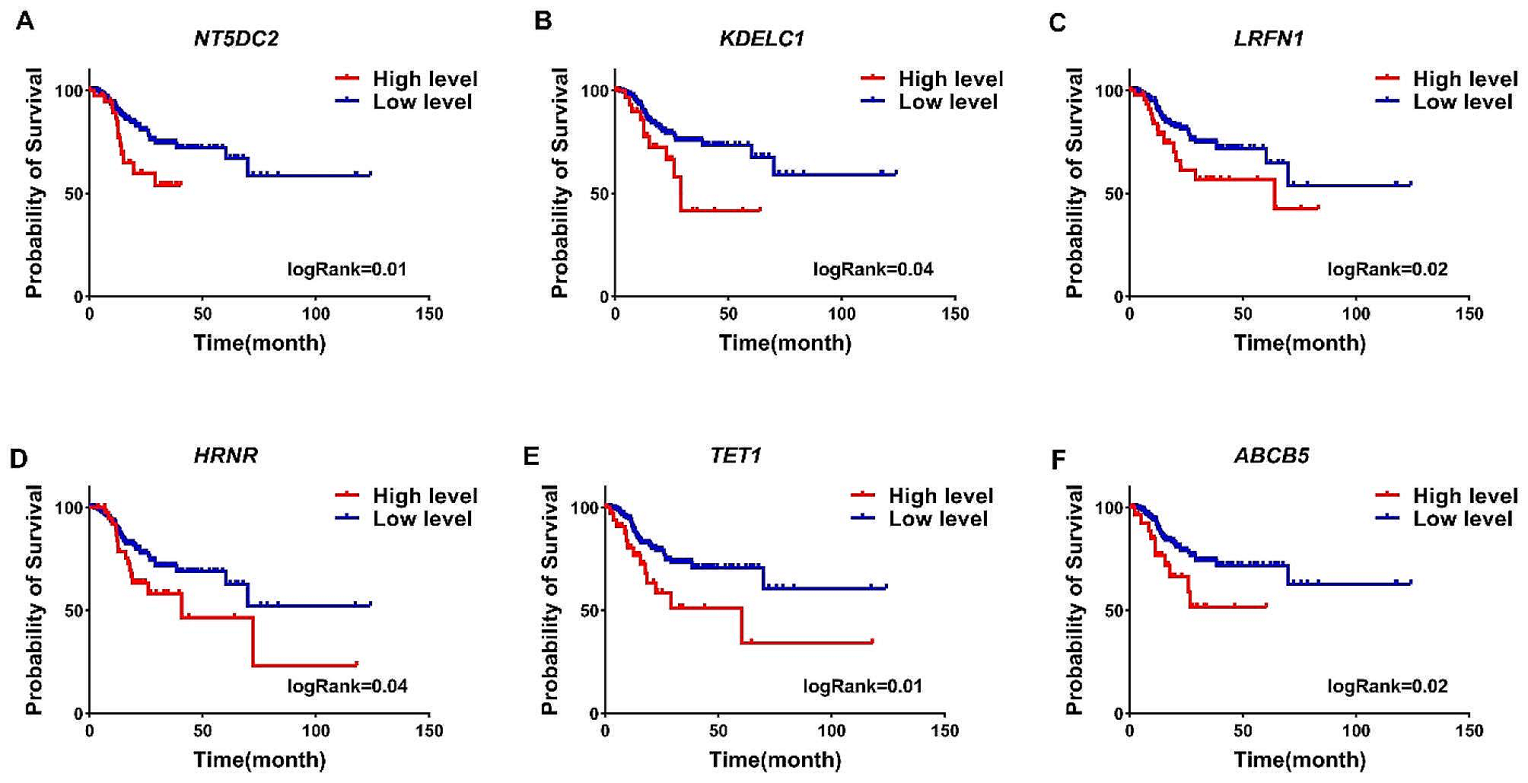
Decreased expression of genes with poor prognosis by EBV. (**A-F**) The genes that exhibit increased expression in GC based on the TCGA data while experiencing decreased expression in EBV-infected GC cell lines, as well as in samples with EBV + status, is shown

**Fig. 4:**
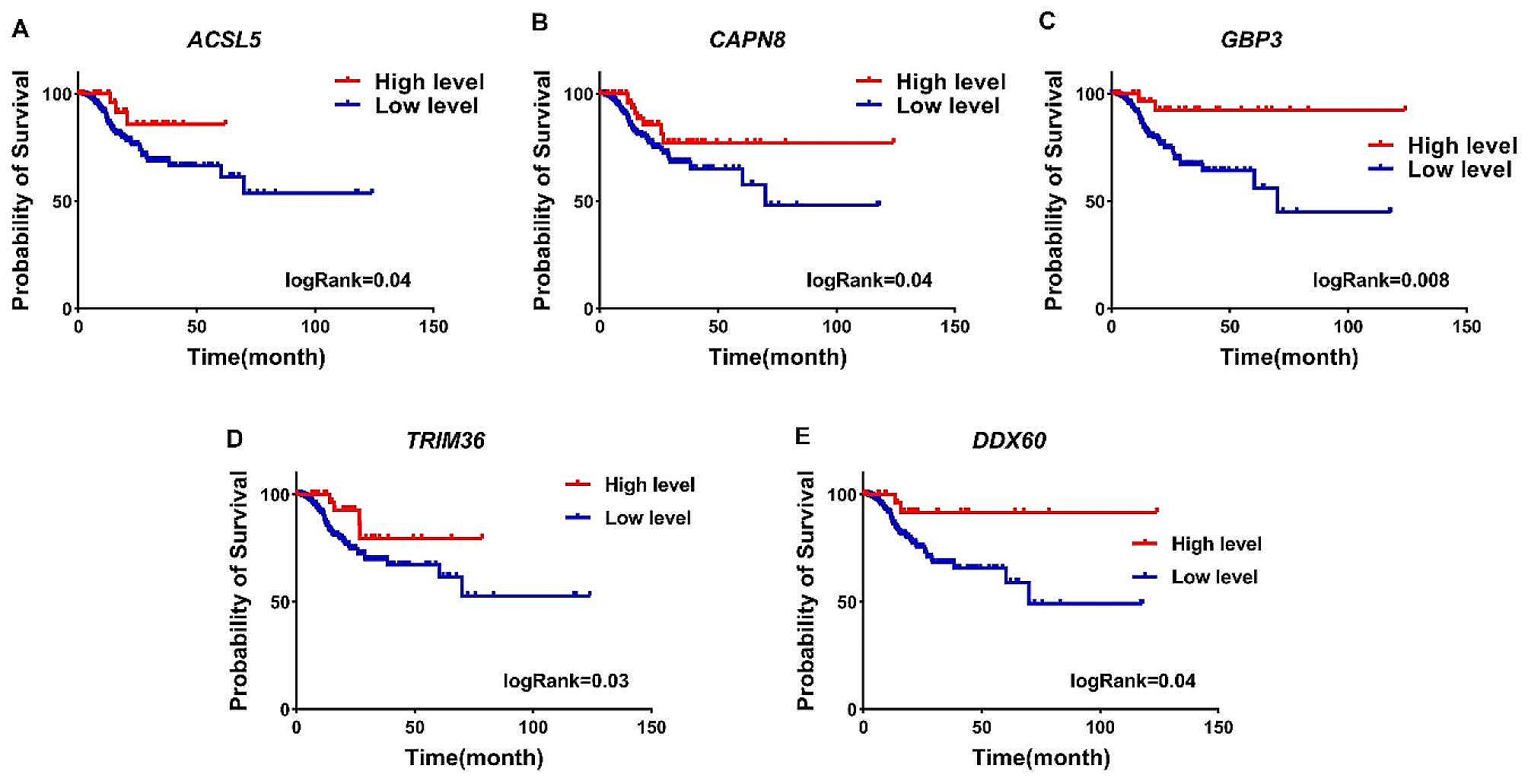
Raised levels of good prognosis genes by EBV. (**A-E**) Genes whose increased expression is associated with good prognosis for patients is pinpointed. Moreover, these genes have the potential to be upregulated by EBV infection

To better understand the impact of EBV on candidate genes and their connection to malignancy and patient mortality, we examined the relationship between their expression levels and patient prognosis using data from TCGA. Our preliminary results showed that increased expression of 6 genes, including *NT5DC2, KDELC1, LRFN1, HRNR, TET1*, and *ABCB5*, is associated with poor prognosis of patients (Table 2), while these 6 genes could decrease expression under the influence of EBV (Supplementary file: Fig. S2 and Table S1). Additionally, Kaplan-Meier (K-M) analysis revealed that elevated expression of these six genes is correlated with a higher mortality rate among patients (Fig. 3A-F, log-rank < 0.05). In contrast, our results showed that the increase of 5 genes including *ACSL5, CAPN8, GBP3, TRIM36*, and *DDX60* are associated with good prognosis of patients (Table 2). In addition, the K-M curve analysis also showed that the increase in the expression of these 5 genes is associated with a decrease in the death rate of patients (Fig. 4A-E, log-rank < 0.05). Our previous findings also indicated that the expression levels of *ACSL5, CAPN8, GBP3, TRIM36*, and *DDX60* could increase with EBV influence, and these genes exhibited higher levels in EBV + samples compared to EBV- (Supplementary file, Table S1 and Fig. S2). These results suggest that EBV may affect genes related to patient survival.

**Table 2:** The univariate Cox regression results for candidate genes based on TCGA data

Gene names	Univariate		
	HR	*P* value	95% CI
*NT5DC2* expression (High vs. Low)	1.32	0.03	1.01– 1.71
*KDELC1* expression (High vs. Low)	1.41	0.01	1.12– 1.86
*LRFN1* expression (High vs. Low)	1.44	0.001	1.16– 1.95
*HRNR* expression (High vs. Low)	1.28	0.01	1.08– 1.69
*TET1* expression (High vs. Low)	1.3	0.01	1.09– 1.69
*ABCB5* expression (High vs. Low)	1.32	0.002	1.09– 1.62
*ACSL5* expression (High vs. Low)	0.73	0.03	0.55– 0.97
*CAPN8* expression (High vs. Low)	0.76	0.02	0.54– 0.96
*GBP3* expression (High vs. Low)	0.72	0.008	0.51– 0.92
*TRIM36* expression (High vs. Low)	0.67	0.007	0.49– 0.91
*DDX60* expression (High vs. Low)	0.71	0.03	0.59– 0.96

### *IFI44L* and *OSA2* genes as hub genes related to EBV-GC axis

**Fig. 5:**
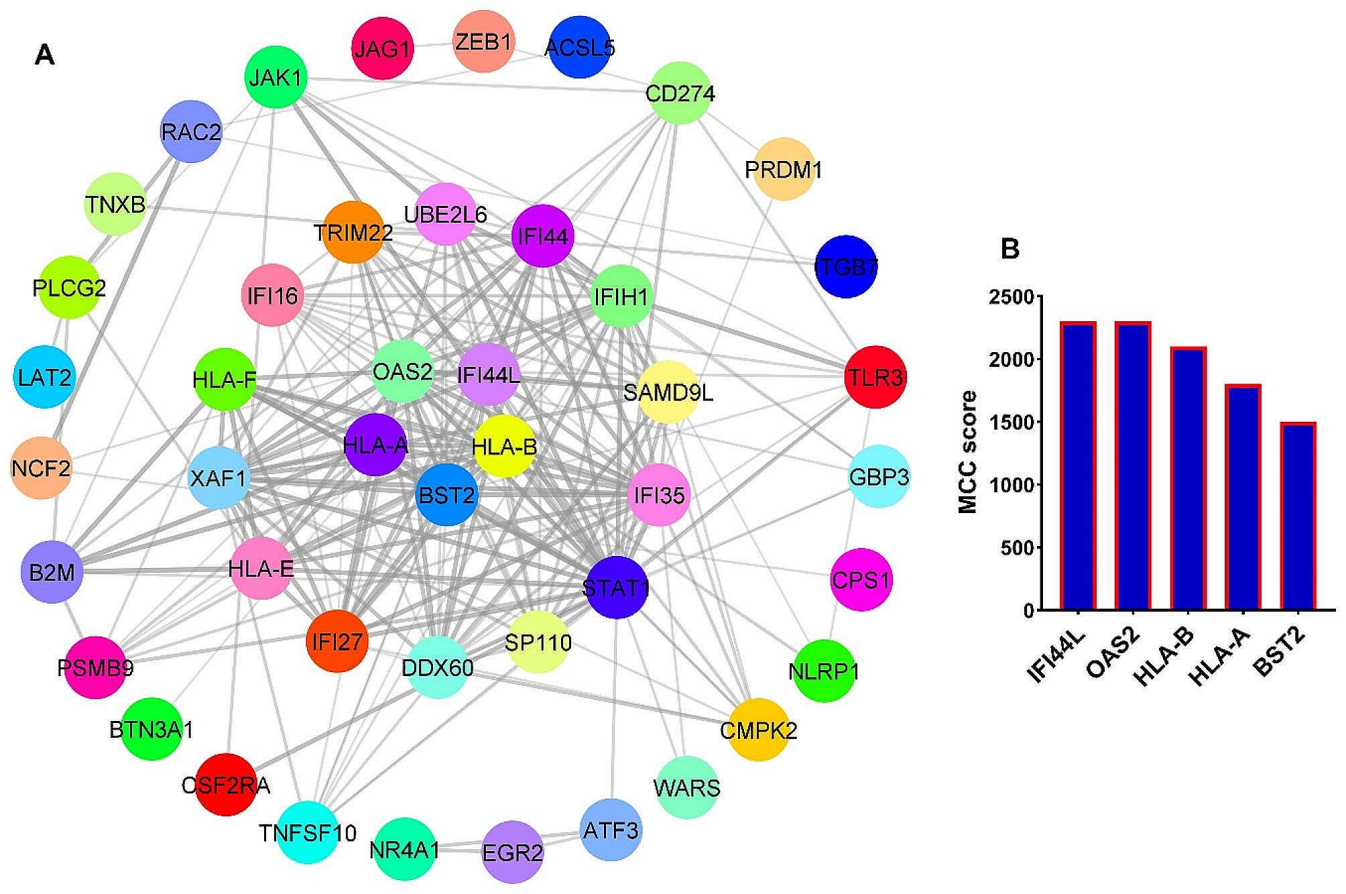
*IFI44L* and *OSA2* genes as hub genes in the EBV-GC axis. (**A**) The PPI network is shown for genes that an increase expression under the influence of EBV and had a higher expression level in EBV + samples compared to EBV- ones (Fig. 2A). (**B**) MCC score for identified hub genes are shown. *IFI44L* and *OSA2* had the highest scores

**Fig. 6:**
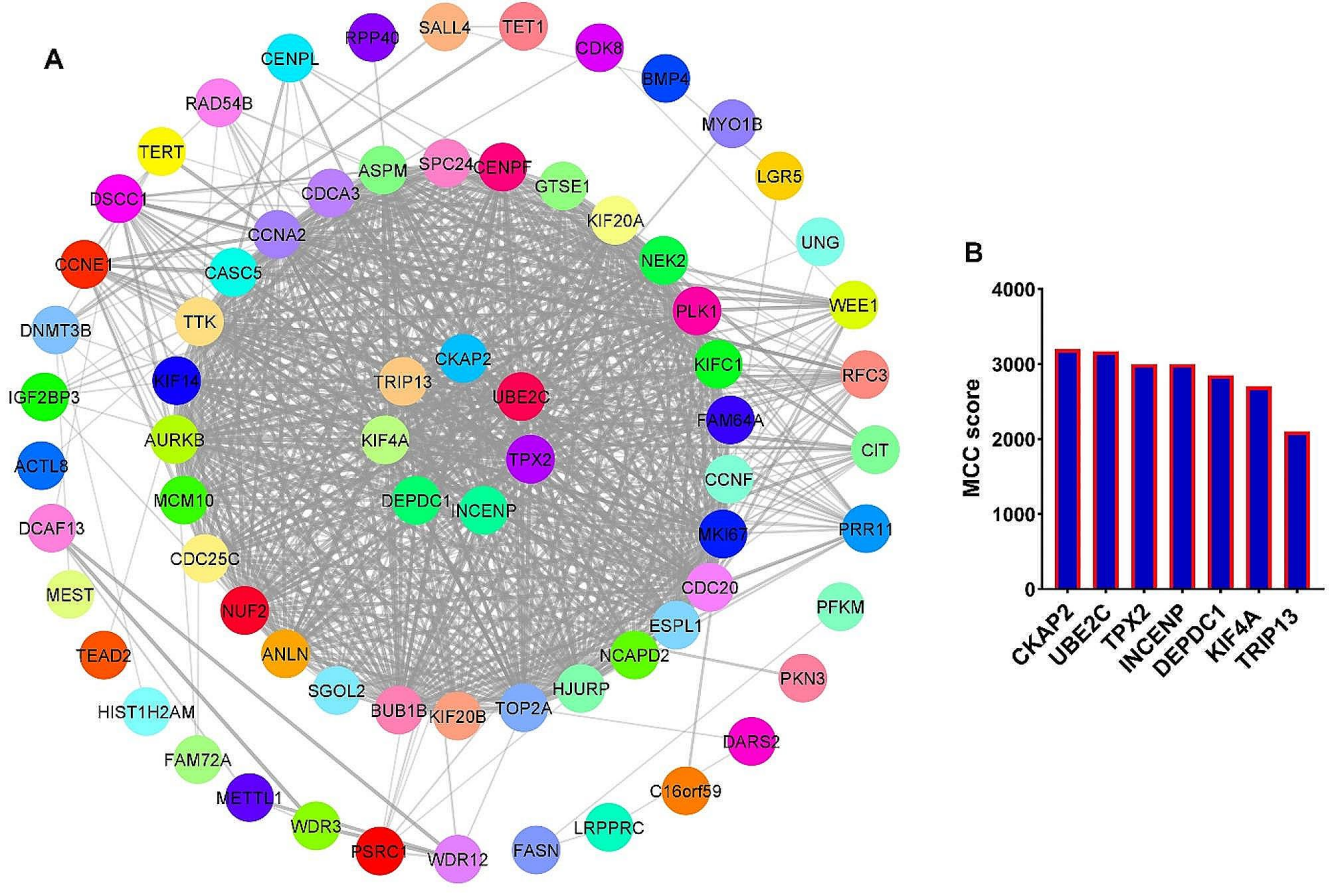
*CKAP2* and *UBE2C* serve as the primary hub genes among those with the potential for reduced expression by EBV. (**A**) PPI network is shown for all genes that have the potential to decrease expression by EBV (Fig. 2A). (**B**) MCC score for identified hub genes are shown. *CKAP2* and *UBE2C* had the highest scores

**Fig. 7:**
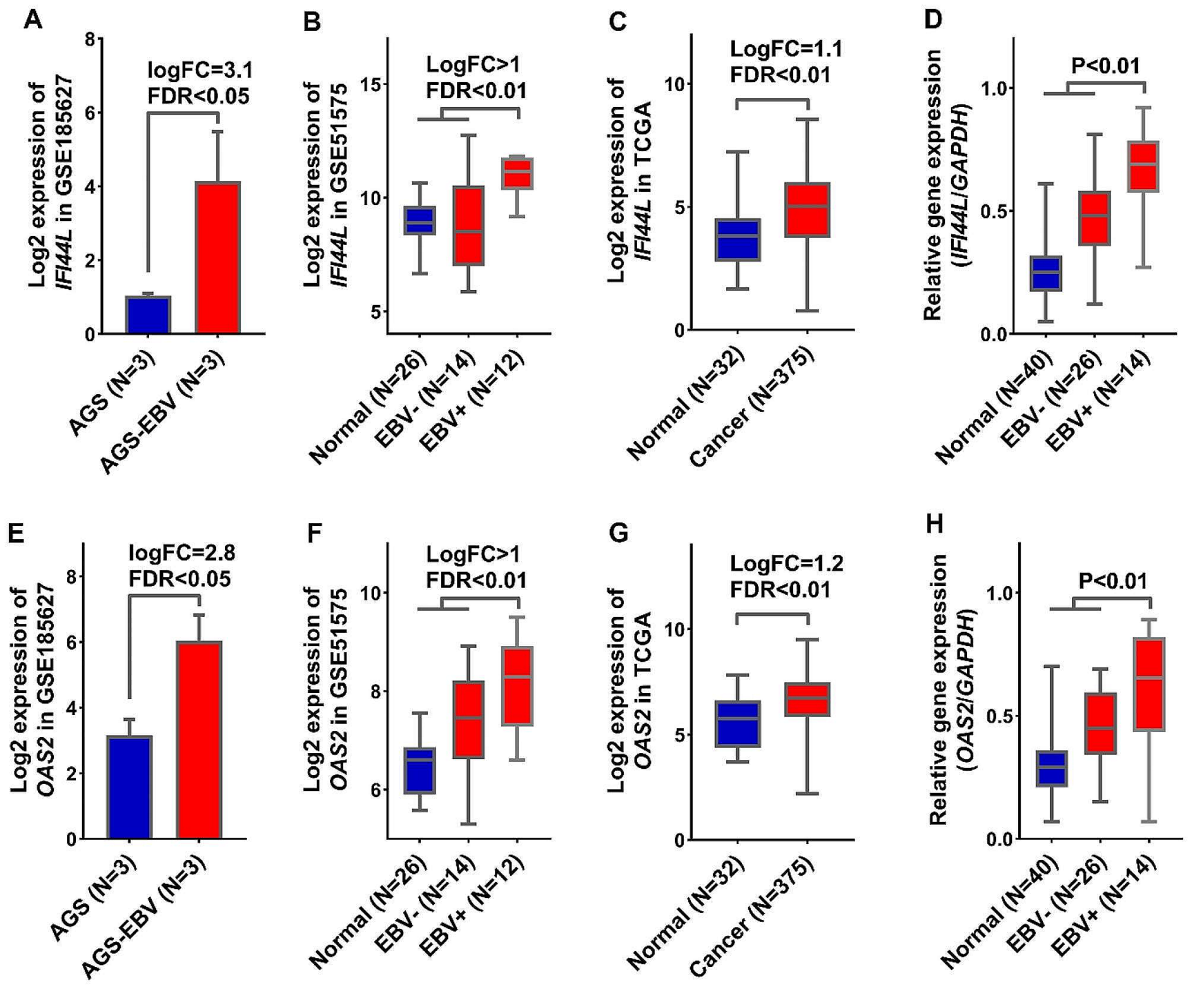
Significant expression changes of *IFI44L* and *OSA2* in EBV + samples compared to normal and EBV- samples. (**A-C**) The alterations in the expression level of *IFI44L* in samples infected with EBV and gastric cancer are depicted based on in silico analyses. (**D**) The RT-qPCR results for the expression level of *IFI44L* in EBV + samples, compared to normal and EBV- samples, is presented. (**E-G**) Based on in silico analysis, the changes in the expression level of *OSA2* in samples infected with the EBV and GC are shown. (**H**) The results of the expression difference for the *OSA2* gene in the studied groups are shown based on the RT-qPCR method

A protein-protein interaction (PPI) network was utilized to identify hubs and significant genes among the candidate genes. To identify hub genes, we used the gene set depicted in Fig. 2A, which was common to both GSE185627 and GSE51575 datasets. The outcomes revealed that out of the 76 increased genes (Fig. 2A), 45 genes can interact with each other (Fig. 5A). In addition, the analysis revealed that five specific genes, namely *IFI44L, OSA2, HLA-B, HLA-A*, and *BST2*, exhibited the highest levels of interaction and MCC scores, solidifying their pivotal roles as hub genes closely associated with EBV (Fig. 5B). Our results showed that among the 107 candidate downregulated genes (Fig. 2A), 71 genes had a PPI network with each other (Fig. 6A). Also, the results of MCODE determined that 7 genes, including *CKAP2, UBE2C, TPX2, INCENP, DEPDC1, KIF4A*, and *TRIP13* can be hub genes related to decreasing genes (Fig. 6B). These findings suggest that the mentioned genes could play a central role in relation to the EBV-GC axis. To confirm the obtained results, the expression level of *IFI44L* and *OSA2* genes in GC samples was investigated by the RT-qPCR method. The reason for choosing these two genes was that both hub genes have been identified from the previous steps, and they play a role in the immune response to the virus. Previous results for *IFI44L* expression changes showed that the expression level of this gene increased significantly in response to EBV infection (Fig. 7A, FDR < 0.01). Also, its expression level increased significantly in EBV + samples compared to EBV- (Fig. 7B, FDR < 0.01) and TCGA data also showed that the expression level of *IFI44L* in cancer samples increased significantly compared to normal (Fig. 7C, FDR < 0.01). The results of RT-qPCR revealed that the expression level of *IFI44L* was significantly higher in EBV + samples compared to normal and EBV- samples (Fig. 7D, *P* < 0.01). On the other hand, our investigations showed that the expression level of *OAS2* could increase in response to EBV (Fig. 7E, FDR < 0.01), and in EBV + samples compared to EBV-, there was a significant increase in expression (Fig. 7F, FDR < 0.01). In addition, TCGA data revealed that the expression level of *OAS2* in cancer samples is significantly increased compared to normal (Fig. 7G, FDR < 0.01). RT-qPCR results showed that EBV samples had significantly higher OAS2 expression levels than normal and EBV- samples (Fig. 7H, *P* < 0.01). These findings confirm the previous results and suggest that *IFI44L* and *OAS2* could be hub genes associated with EBV in GC.

## Discussion

It has been shown that different factors, including the infection of gastric epithelial cells with EBV, can play a role in the development and malignancy of GC [16]. Different studies have determined that EBV can be involved in GC malignancy through molecular changes such as mutation and epigenetic changes [17, 18]. The relationship between EBV and transcriptomic changes in GC has been less investigated, and the impact of gene expression changes on patient mortality rates remains an understudied aspect of this field. In this study, we utilized in silico data along with EBV + cancer samples to identify genes with EBV-influenced expression changes related to GC malignancy and provided an overview of EBV transcriptomic alterations in this context.

Our preliminary results showed that many genes affected by EBV infection change their expression, and these genes play a role in the main pathways of cancer cells. Investigations showed that many of the genes that could be increased under the influence of EBV are involved in pathways such as immune responses to the virus, TP53, and inflammation. Furthermore, our analyses indicated that in samples with EBV + status, the expression levels of genes associated with these pathways were significantly higher compared to EBV-samples. Extensive evidence shows that one of the main mechanisms associated with EBV in the development of GC is inflammation [19]. Inflammation can result from various inflammatory mediators, including cytokines, chemokines, prostaglandins, growth and transcription factors, microRNAs, and enzymes like cyclooxygenase and matrix metalloproteinase. Together, these components collaborate to establish a conducive microenvironment for tumor development [20]. Additionally, inflammation leads to elevated levels of free radicals, thereby increasing DNA mutation rates and the risk of cancer. This surge in free radicals and DNA damage triggers the activation of the P53 pathway [21, 22]. The results of TCGA data also showed that the genes related to inflammation are increased in cancer samples compared to normal. These findings show that inflammatory pathways and the increase of free radicals in response to EBV infection could play a role in increasing the risk of GC. Our results also suggest that EBV may increase the expression of genes associated with the hypoxia pathway in cellular models. However, other findings indicate that genes related to this pathway were lower in EBV + samples compared to EBV- samples. Further investigations have demonstrated that certain EBV membrane proteins could induce key factors associated with hypoxia, such as HIF1α, triggering this pathway in cellular models [23]. For EBV to enter the lytic phase, it requires involvement with the hypoxia pathways [24]. The downregulation of genes associated with the hypoxia pathway in EBV + samples seems to be connected to the virus’s replication cycle. This underscores the necessity for additional studies to elucidate the relationship between EBV and the hypoxia pathways.

We identified 107 genes that showed a significant decrease in expression in GC cell lines under the influence of EBV infection. These genes exhibited lower expression levels in EBV + samples compared to EBV-. Enrichment results for these 107 genes revealed that many of them are involved in cell proliferation pathway such as E2F Targets, Myc Targets, Mitotic Spindle, and G2-M Checkpoint. The investigations have shown that AGS cell lines infected by EBV exhibit reduced growth and migration capabilities compared to the control group [25]. Moreover, it has been demonstrated that certain microRNAs generated by EBV, such as miR-BART12, can target crucial genes involved in cellular proliferation and migration pathways [26]. It has been demonstrated that AGS cells infected with EBV exhibit a significantly reduced rate of cellular proliferation compared to non-infected cells [25]. On the other hand, our results showed that the increased expression of some genes, such as *NT5DC2, KDELC1, LRFN1, HRNR, TET1*, and *ABCB5* in GC is associated with a poor prognosis for patients, while the expression of the mentioned genes could be reduced by EBV. It has been reported that increased expression of *NT5DC2* is associated with poor prognosis for GC patients, and its expression might be higher in metastatic samples [27]. The roles of other mentioned genes and their comprehensive association with GC and EBV are yet to be fully understood, and in this study, we report their findings for the first time. Also, increased expression of genes such as *ACSL5, CAPN8, GBP3, TRIM36*, and *DDX60* is associated with a good prognosis of patients, and their expression could be increased by EBV. Studies have also shown that EBV + samples have a better prognosis compared to EBV- samples [4]. These findings suggest that at the transcriptomic level, EBV has the potential to modulate genes associated with malignancy in cells.

Candidate genes and the PPI network were used to identify hub genes related to the EBV-GC axis. The results showed that five genes, including *IFI44L, OSA2, HLA-B, HLA-A*, and *BST2* can have a central role in the increased genes affected by EBV. RT-qPCR results showed that the expression level of *IFI44L* and *OSA2* was higher in EBV + samples compared to normal and EBV- samples. *IFI44L* and *OSA2* are two genes that mediate the immune response to viruses through interferon [28]. Studies have shown that these two genes can play a role in drug resistance to Trastuzumab in GC and thus play a role in GC malignancy [29]. It has also been shown that the expression level of *IFI44L* in GC can help distinguish different subgroups of GC [30]. Our results suggest that the expression level of *IFI44L* and OSA2 could be higher in the subgroup of GC with EBV + status. These genes may be considered potential therapeutic targets in this specific subgroup. In general, the results of this study showed that EBV could increase the risk of GC through inflammatory pathways. It can modulate genes related to cell proliferation and genes related to patient survival rate and provide better survival for GC patients. These results require more in-vitro and in-vivo studies to clarify the role of EBV in GC, and this was one of the main limitations of this study.

## Conclusion

The overall findings of this study suggest that EBV may downregulate certain genes associated with malignancy pathways and adverse prognosis at the RNA level. Conversely, EBV-associated upregulated genes are involved in pathways such as inflammation. Therefore, the role of EBV in GC pathogenesis could be dual-sided. One hypothesis that can be inferred is that EBV might enhance the risk of GC through inflammation and other reported pathways in the early stages. However, in cancer cells, the presence of EBV may oppose pathways associated with malignancy, such as cellular proliferation and migration, as indicated by our results and supported by other studies.

### Electronic supplementary material

Below is the link to the electronic supplementary material.

Supplementary Material 1

## Data Availability

Supporting and raw data are available upon a reasonable request to the corresponding author.

## Abbreviations

GC: Gastric cancer
EBV: Epstein-Barr virus
EBV+: EBV-positive
EBV-: EBV-negative
TCGA: The cancer genome atlas
K-M: Kaplan-Meier
PPI: Protein-protein interaction
GEO: Gene Expression Omnibus
