# Supplementary material for: Recognizing the role of Epstein-Barr virus in gastric cancer: transcriptomic insights into malignancy modulation
Source: Virol J. 2024 Feb 14;21:41. doi: 10.1186/s12985-024-02307-z (PMC10868016; doi:10.1186/s12985-024-02307-z)
Supplement: Supplementary file 1 — Supplementary Material 1 [file 12985_2024_2307_MOESM1_ESM.docx]

**Table S1:** The list of candidate genes that could be significantly changed under the influence of EBV in both GSE185627 and GSE51575 studies is provided.

| **Candidate Genes** | **logFC** | **Adj.P.Val** | **Candidate Genes** | **logFC** | **Adj.P.Val** |
| --- | --- | --- | --- | --- | --- |
| TPX2 | -1.19531 | 0.028055 | SGOL2 | -1.13128 | 0.009308 |
| KIF20A | -1.66049 | 0.000994 | KDELC1 | -1.07806 | 0.00293 |
| ESPL1 | -1.23604 | 0.015496 | CENPL | -1.1457 | 0.003693 |
| TOP2A | -1.32678 | 0.025253 | DEPDC1 | -1.20518 | 0.008145 |
| CDC20 | -1.14847 | 0.01578 | BMP4 | -2.62429 | 0.000102 |
| SALL4 | -2.391 | 0.003801 | FASN | -1.59393 | 1.82E-06 |
| FAM64A | -2.05434 | 8.05E-05 | NCAPD2 | -1.24574 | 0.000352 |
| GTSE1 | -1.04956 | 0.016659 | PSRC1 | -1.02053 | 0.008732 |
| CDCA3 | -1.26112 | 0.004907 | METTL1 | -1.06475 | 1.15E-05 |
| C16orf59 | -1.28409 | 0.005895 | LRFN1 | -1.53322 | 0.001324 |
| ASPM | -1.10387 | 0.0303 | CCDC138 | -1.09175 | 0.002214 |
| CNTNAP2 | -1.90465 | 0.002039 | GGT7 | -1.20076 | 0.0022 |
| PYCRL | -1.18961 | 3.48E-05 | PDSS1 | -1.08859 | 0.010052 |
| KIF4A | -1.81607 | 0.029731 | LAPTM4B | -1.85608 | 0.001821 |
| CCNA2 | -1.18922 | 0.012371 | ANLN | -1.18468 | 0.031976 |
| NT5DC2 | -1.26801 | 0.001848 | WEE1 | -1.05367 | 0.001116 |
| CASC5 | -1.5621 | 0.037042 | KIF20B | -1.00053 | 0.004158 |
| DNMT3B | -1.26262 | 0.041628 | DCAF13 | -1.1181 | 0.000688 |
| CIT | -1.16557 | 0.000525 | NUF2 | -1.28727 | 0.003628 |
| ACTL8 | -1.59079 | 0.004354 | KIFC1 | -1.18406 | 0.026263 |
| TRIP13 | -1.14163 | 0.002091 | UBE2C | -1.21048 | 0.041249 |
| SCARB1 | -1.33003 | 0.000248 | PLK1 | -1.37589 | 0.00522 |
| GRB14 | -1.64905 | 0.015819 | HRNR | -1.07484 | 0.011155 |
| PROCR | -1.35516 | 0.004491 | PRR11 | -1.08036 | 0.006965 |
| LRP4 | -1.88245 | 0.002088 | GAS2L1 | -1.02079 | 0.008335 |
| RPP40 | -1.22008 | 9.77E-06 | TEAD2 | -1.08501 | 0.003174 |
| CENPF | -1.14499 | 0.002802 | UCK2 | -1.11794 | 1.21E-05 |
| CLCN2 | -1.27135 | 0.006368 | TERT | -1.27509 | 0.002302 |
| INCENP | -1.06072 | 0.002659 | CCNE1 | -2.00782 | 2.27E-06 |
| KIF14 | -1.05289 | 0.041441 | DBNDD1 | -1.69159 | 0.00026 |
| CCNF | -1.00258 | 0.004563 | ADORA1 | -1.07487 | 0.018698 |
| BUB1B | -1.15717 | 0.032796 | PGAM5 | -1.0464 | 0.001781 |
| CDC25C | -1.0592 | 0.016998 | VIPR1 | -1.79386 | 0.004515 |
| C17orf96 | -1.61255 | 0.000406 | HJURP | -1.14147 | 0.046247 |
| MEST | -1.1791 | 0.010451 | DHRS13 | -1.0029 | 0.008396 |
| APOA2 | -2.15883 | 0.027065 | CHML | -1.32481 | 2.95E-05 |
| LGR5 | -1.49418 | 0.019359 | TET1 | -1.33529 | 0.022367 |
| RFC3 | -1.31514 | 0.002088 | TMEM141 | -1.77502 | 7.93E-07 |
| IGF2BP3 | -1.69707 | 0.014741 | DARS2 | -1.36188 | 2.25E-05 |
| PKN3 | -1.1343 | 0.002451 | AURKB | -1.24469 | 0.010562 |
| UNG | -1.4065 | 6.49E-07 | ZNF614 | -1.47434 | 0.000626 |
| LRPPRC | -1.02479 | 6.96E-05 | CREG2 | -1.22632 | 0.009422 |
| DSCC1 | -1.42359 | 0.000557 | GJD3 | 1.026784 | 0.032191 |
| PFKM | -1.34427 | 1.91E-05 | IFI44 | 2.013702 | 8.17E-05 |
| LOC730101 | -1.12585 | 0.001959 | IFIH1 | 1.370104 | 2.31E-05 |
| MCM10 | -1.2695 | 0.001664 | OAS2 | 2.075456 | 2.31E-05 |
| ARSE | -1.61375 | 0.028125 | WARS | 2.529046 | 1.74E-05 |
| ZNF761 | -1.07905 | 0.001575 | CPS1 | 2.906935 | 0.049162 |
| NEK2 | -1.53214 | 0.001008 | SEL1L | 1.049361 | 5.02E-05 |
| FAM72A | -1.45854 | 0.001363 | IFI35 | 1.068111 | 0.009956 |
| ACACA | -1.05737 | 8.55E-06 | PDE4D | 1.040711 | 0.007986 |
| SACS | -1.5636 | 4.06E-05 | EGR2 | 1.507618 | 0.009681 |
| MKI67 | -1.34555 | 0.007275 | IFI27 | 1.906173 | 0.004421 |
| HIST1H2AM | -1.53009 | 0.018753 | SAT1 | 1.16495 | 0.004495 |
| CKAP2 | -1.05024 | 0.001823 | LAT2 | 1.593871 | 0.000441 |
| OPN3 | -1.11397 | 8.22E-05 | UBE2L6 | 1.581859 | 0.000136 |
| TMEM117 | -1.15826 | 0.000435 | BST2 | 1.836621 | 0.002749 |
| TTK | -1.27101 | 0.001747 | F8 | 1.784184 | 4.15E-05 |
| ABCB5 | -1.0896 | 0.005386 | STAT1 | 1.982145 | 0.00025 |
| CDK8 | -1.06246 | 0.000127 | GBP3 | 1.446294 | 0.048227 |
| RAD54B | -1.22664 | 0.001468 | TMEM30B | 1.277917 | 0.013831 |
| WDR12 | -1.10612 | 1.36E-05 | ZEB1 | 1.091573 | 0.002891 |
| SPC24 | -1.08194 | 0.002207 | IFI44L | 2.148166 | 0.002031 |
| MYO1B | -1.3387 | 1.25E-05 | JAG1 | 1.255079 | 0.015751 |
| WDR3 | -1.2495 | 5.64E-05 | HLA-B | 1.493501 | 0.00024 |
| SECTM1 | 1.609217 | 0.010348 | MIA | 4.627019 | 0.000255 |
| ABCC3 | 1.323414 | 0.008882 | HLA-F | 2.250592 | 2.58E-06 |
| LAMP3 | 2.020462 | 0.000424 | PLCG2 | 1.2794 | 0.001681 |
| PRDM1 | 1.668219 | 0.000702 | ITGB7 | 1.750285 | 0.000102 |
| SERPING1 | 1.373274 | 0.002234 | DTNA | 1.206529 | 5.72E-05 |
| ANKRD12 | 1.384176 | 0.002697 | NLRP1 | 1.024346 | 0.000805 |
| LOC728392 | 1.234097 | 0.0032 | BTN3A1 | 1.589865 | 0.000136 |
| ACSL5 | 1.177968 | 0.04309 | CMPK2 | 1.220142 | 0.016189 |
| CA2 | 3.221579 | 0.000164 | B2M | 2.132462 | 1.21E-05 |
| IFI16 | 1.614326 | 0.002007 | SAMD9L | 2.445786 | 8.10E-05 |
| CD274 | 1.968442 | 0.005139 | GAA | 1.584348 | 5.93E-05 |
| LMO2 | 1.017744 | 0.033255 | OPTN | 1.045473 | 0.001013 |
| SLC28A3 | 1.195097 | 0.026393 | PSMB9 | 1.649961 | 0.000709 |
| CTSO | 1.544789 | 0.000438 | ODF3B | 1.285969 | 0.004233 |
| CLIP4 | 1.052798 | 0.036763 | JAK1 | 1.081344 | 0.00236 |
| CST7 | 1.197312 | 0.005467 | NR4A1 | 1.413427 | 0.024676 |
| CAPN8 | 2.338594 | 0.00278 | CSF2RA | 1.207187 | 0.000335 |
| TNFSF10 | 1.315195 | 0.006396 |  |  |  |
| TRIM22 | 2.788045 | 9.88E-07 |  |  |  |
| SP110 | 1.076261 | 0.001023 |  |  |  |
| SLC8A1 | 2.020137 | 1.55E-06 |  |  |  |
| RASIP1 | 1.033067 | 0.015775 |  |  |  |
| TRIM36 | 1.176522 | 0.017634 |  |  |  |
| HLA-A | 1.849265 | 2.18E-05 |  |  |  |
| RAC2 | 1.523029 | 0.000357 |  |  |  |
| TLR3 | 1.174883 | 0.043709 |  |  |  |
| DDX60 | 1.446514 | 0.027814 |  |  |  |
| TNXB | 1.240784 | 0.010985 |  |  |  |
| TMED7-TICAM2 | 1.070605 | 0.007513 |  |  |  |
| FAM49A | 1.227948 | 0.003283 |  |  |  |
| XAF1 | 1.889664 | 2.66E-05 |  |  |  |
| HAP1 | 1.120468 | 8.02E-05 |  |  |  |
| ATF3 | 1.179061 | 0.026156 |  |  |  |


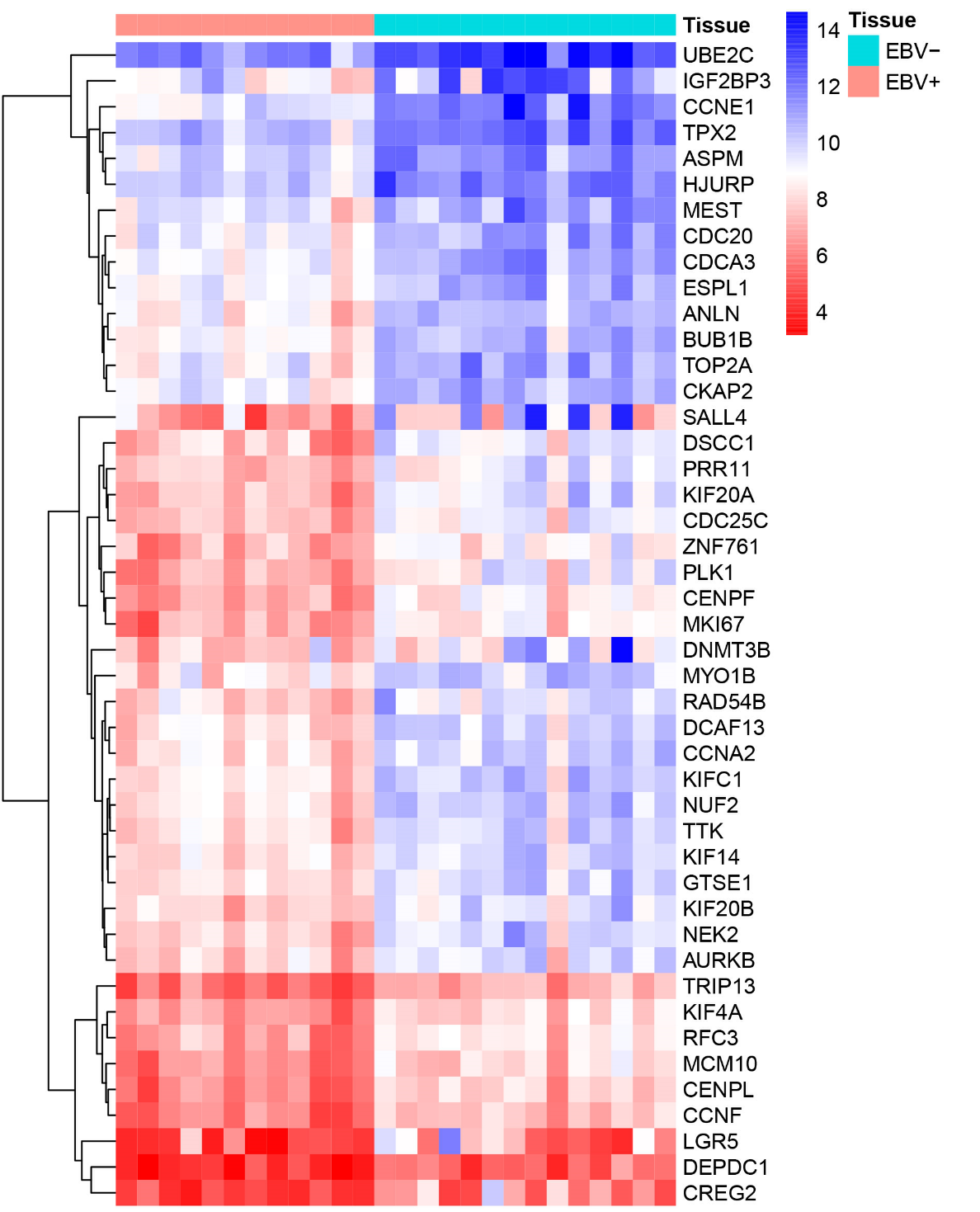


**Fig. S1: Downregulated genes in EBV+ samples.** Heat map for genes that were increased in cancer samples compared to normal based on TCGA data, while they had a significant decrease in expression in EBV+ samples compared to EBV- samples based on GSE51575.


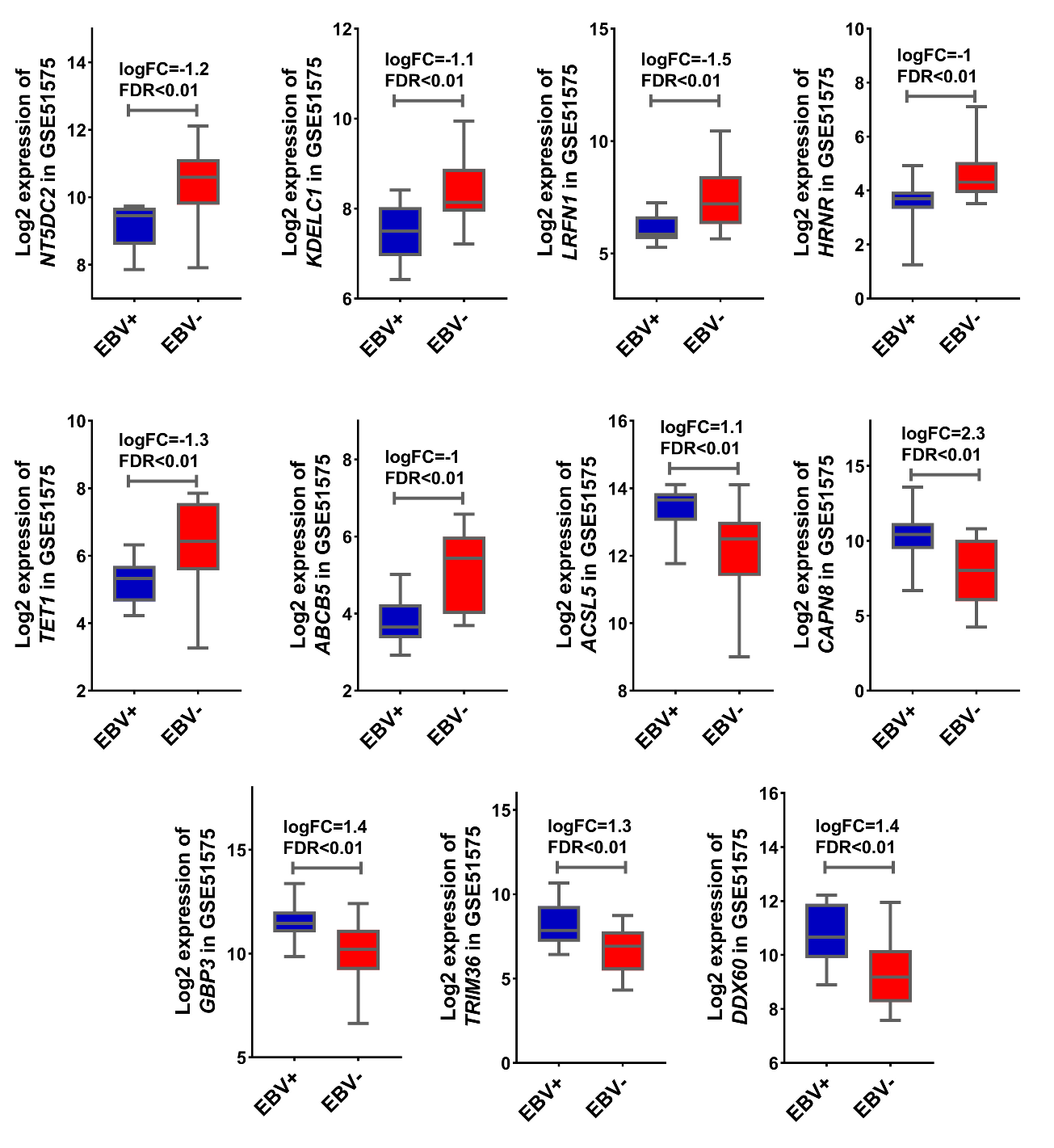


**Fig. S2: Changes in gene expression related to the survival rate of patients in the presence of EBV.** The expression levels of genes associated with the survival rate of patients, based on TCGA data, are shown in EBV-positive samples.
